# Absence of Staphylococcus aureus in Wild Populations of Fish Supports a Spillover Hypothesis

**DOI:** 10.1128/spectrum.04858-22

**Published:** 2023-06-21

**Authors:** Marta Matuszewska, Alicja Dabrowska, Gemma G. R. Murray, Steve M. Kett, Andy J. A. Vick, Sofie C. Banister, Leonardo Pantoja Munoz, Peter Cunningham, John J. Welch, Mark A. Holmes, Lucy A. Weinert

**Affiliations:** a Department of Veterinary Medicine, University of Cambridge, Cambridge, United Kingdom; b Department of Medicine, University of Cambridge, Cambridge, United Kingdom; c Department of Physics, University of Cambridge, Cambridge, United Kingdom; d Department of Genetics, Evolution and Environment, University College London, London; e Department of Natural Sciences, Middlesex University London, London, United Kingdom; f Wester Ross Fisheries Trust, Harbour Centre, Gairloch, Wester Ross, United Kingdom; g RAL Space (UKRI-STFC), Harwell Campus, Didcot, Oxfordshire, United Kingdom; h School of History, Classics and Archaeology, University of Edinburgh, Edinburgh, United Kingdom; i Department of Genetics, University of Cambridge, Cambridge, United Kingdom; USGS, Eastern Ecological Science Center

**Keywords:** MRSA, brown trout, spillover, Scottish Highlands, host range

## Abstract

Staphylococcus aureus is a human commensal and opportunistic pathogen that also infects other animals. In humans and livestock, where S. aureus is most studied, strains are specialized for different host species. Recent studies have also found S. aureus in diverse wild animals. However, it remains unclear whether these isolates are also specialized for their hosts or whether their presence is due to repeated spillovers from source populations. This study focuses on S. aureus in fish, testing the spillover hypothesis in two ways. First, we examined 12 S. aureus isolates obtained from the internal and external organs of a farmed fish. While all isolates were from clonal complex 45, genomic diversity indicates repeated acquisition. The presence of a φSa3 prophage containing human immune evasion genes suggests that the source was originally human. Second, we tested for S. aureus in wild fish that were isolated from likely sources. In particular, we sampled 123 brown trout and their environment at 16 sites in the remote Scottish Highlands with variable levels of exposure to humans, birds, and livestock. This screen found no S. aureus infection in any of the wild populations or their environment. Together, these results support that the presence of S. aureus in fish and aquaculture is due to spillover from humans rather than specialization. Given the trends of increasing fish consumption, a better understanding of the dynamics of S. aureus spillover in aquaculture will mitigate future risks to fish and human health.

**IMPORTANCE** Staphylococcus aureus is a human and livestock commensal but also an important pathogen responsible for high human mortality rates and economic losses in farming. Recent studies show that S. aureus is common in wild animals, including fish. However, we do not know whether these animals are part of the normal host range of S. aureus or whether infection is due to repeated spillover events from true S. aureus hosts. Answering this question has implications for public health and conservation. We find support for the spillover hypothesis by combining genome sequencing of S. aureus isolates from farmed fish and screens for S. aureus in isolated wild populations. The results imply that fish are unlikely to be a source of novel emergent S. aureus strains but highlight the prominence of the spillover of antibiotic-resistant bacteria from humans and livestock. This may affect both future fish disease potential and the risk of human food poisoning.

## INTRODUCTION

Staphylococcus aureus is both a common commensal bacterium of the human nasopharynx and skin and an opportunistic pathogen (1). S. aureus also colonizes and causes infections in companion animals (2, 3) and livestock (4, 5), with the latter resulting in significant morbidity and economic losses (6). Recent studies have shown that major human and livestock strains, which are distinguished by a profile of housekeeping genes and named clonal complexes (CCs), are also found in many wild animal species (7–12). However, little is known about whether they are a consequence of recent spillovers from human and/or livestock populations (i.e., the limited transmission of a strain in a novel host group with no evidence of adaptation) or if they persist independently within wild animal species. These two scenarios have different implications for both public health and conservation. For spillovers, humans and livestock are major sources of environmental contamination with a bacterium that is capable of causing disease in diverse host species and is often resistant to antibiotics. Persistent carriage of or infection by novel emergent strains of S. aureus in wild animal species may pose a public health risk to human and livestock health (13–16). To distinguish between these two possibilities, genomic data from isolates from wild animal populations can be used to infer transmission dates and routes and to identify genetic changes that may represent host-specific adaptation (5, 17–25).

S. aureus, including methicillin-resistant S. aureus (MRSA), is frequently reported in fish and fishery products, with prevalences ranging from 2 to 60% (26). The presence of S. aureus in fish is concerning because the ingestion of staphylococcal enterotoxins produced by S. aureus can cause food poisoning (27, 28). It is widely assumed that the presence of S. aureus in fish products is a result of contamination during handling and processing (29). However, the combined use of antimicrobial drugs in aquaculture and the subsequent contamination of aquatic environments could contribute to the selection, emergence, and spread of antibiotic-resistant S. aureus (30, 31). Supporting this conjecture, S. aureus has also been found in fish sampled directly from aquaculture, where fish processing and handling are limited, and MRSA strains have been identified in cage-cultured tilapia in Malaysia (32), tank-cultured dusky kob in South Africa (33), and farmed fish in Iran (34). Only one study, by Salgueiro et al., performed genome sequencing and found CC398 methicillin-sensitive S. aureus (MSSA) (35), most likely human associated (22, 36, 37). That study suggested that S. aureus is present in fish due to spillover from human populations but did not have the discrimination to test whether S. aureus can adapt to and persist within fish populations or aquatic environments.

In this study, we investigate whether S. aureus is present in fish from potential source populations in the Scottish Highlands. The Scottish Highlands are among the least densely populated regions of Europe, with an average density of 8 persons per km^2^ (38). We sampled wild brown trout (Salmo trutta L. 1758) and their environment, including water and sediment. Brown trout vary in their habitats, ranging from highland streams to arctic fjords (39), which allowed us to investigate the prevalence of S. aureus in areas with various levels of exposure to known hosts of S. aureus. We obtained samples from lochs close to livestock, lochs within bird-roosting/feeding areas, sea sites close to human populations, and remote lochs that are isolated from human, bird, and livestock populations. While we readily detected S. aureus from multiple organs of a farmed fish in London, England, we did not detect S. aureus in any Scottish trout or their environment. These results support that the presence of S. aureus in farmed fish is due to recent spillover from other host populations.

## RESULTS

### S. aureus sample calibration and whole-genome diversity from a farmed fish.

To establish the most effective method for detecting S. aureus from fish, we swabbed and homogenized tissue from different body sites of a farmed rainbow trout (Oncorhynchus mykiss). S. aureus was successfully isolated from all sites apart from a heart tissue sample and a mouth swab sample (Table 1). All positive tissue samples were also detected using charcoal swabs, suggesting that tissue processing is unnecessary for S. aureus detection from positive sites. Both swabs from researchers carrying out the sample processing were negative for S. aureus. All of the trout, whether wild or farmed, that were collected for this study appeared to be healthy and did not exhibit any signs of disease except for a few individuals that had a low level of diplostomiasis. However, these infection levels were deemed to be insignificant and were not expected to affect the normal behavior of the trout.

**TABLE 1:** Detection of S. aureus from a single rainbow trout purchased from a fish farm in London, England

Sampling method	Sampling site	Result	
		S. aureus detection using selective plates	Detection of the *femB* gene by PCR
Charcoal swab	Mouth	−	−
	Gills	+	+
	Skin	+	+
	Vent	+	+
Tissue	Intestine	+	+
	Gills	+	+
	Skin	+	+
	Heart	−	−
Charcoal swab	Researcher 1 (nasal)	−	−
Charcoal swab	Researcher 2 (nasal)	−	−

**FIG 1:**
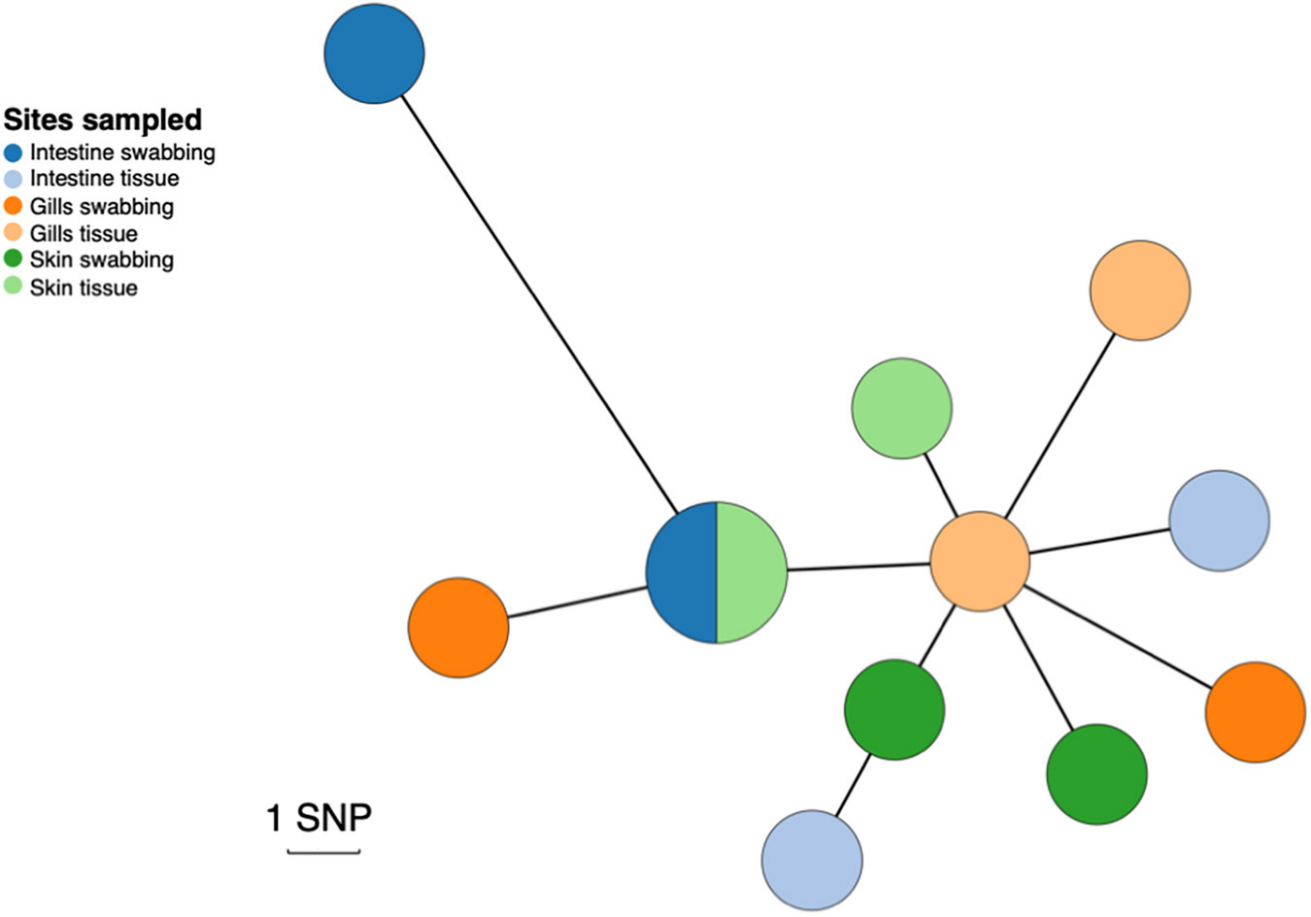
Genetic diversity of S. aureus isolates obtained from three different anatomic sites in a single farmed rainbow trout from a London fish farm. A minimum-spanning tree was constructed using an alignment of the 12 fish isolates, created by mapping to the S. aureus LGA251 reference genome. Points represent groups of identical isolates, with point sizes correlated to the number of isolates. Due to the low number of SNPs, each SNP was manually checked to ensure that it was not a consequence of mapping errors. The colors represent different sampling fish sites: intestine, gills, and skin. Isolates extracted using charcoal swabs are indicated in a darker shade, whereas isolates extracted from tissue samples are indicated in a lighter shade.

To investigate within-fish diversity, whole-genome sequencing was carried out for 12 S. aureus isolates obtained from different body sites. All isolates were identified as strain type 54 (ST54), which is part of clonal complex 45 (CC45). Figure 1 shows a grape plot from a core-genome alignment mapped to the S. aureus LGA251 reference genome. The isolates are not clustered by sampling site (intestine, gill, and skin) or by sampling method (charcoal swabbing and tissue sampling). The isolates are very closely related, with the maximum distance between two isolates being 12 single nucleotide polymorphisms (SNPs). Based on an SNP clock rate of ~3.5 SNPs/genome/year for ST22 (40), we estimated that these isolates’ most recent common ancestor would have dated to around 2017 (2 years before sampling). This was confirmed using a dated phylogeny where an estimate of the clade origin was approximately 2017 (1.5 years before sampling) (95% confidence interval [CI], 2016 to 2018).

**FIG 2:**
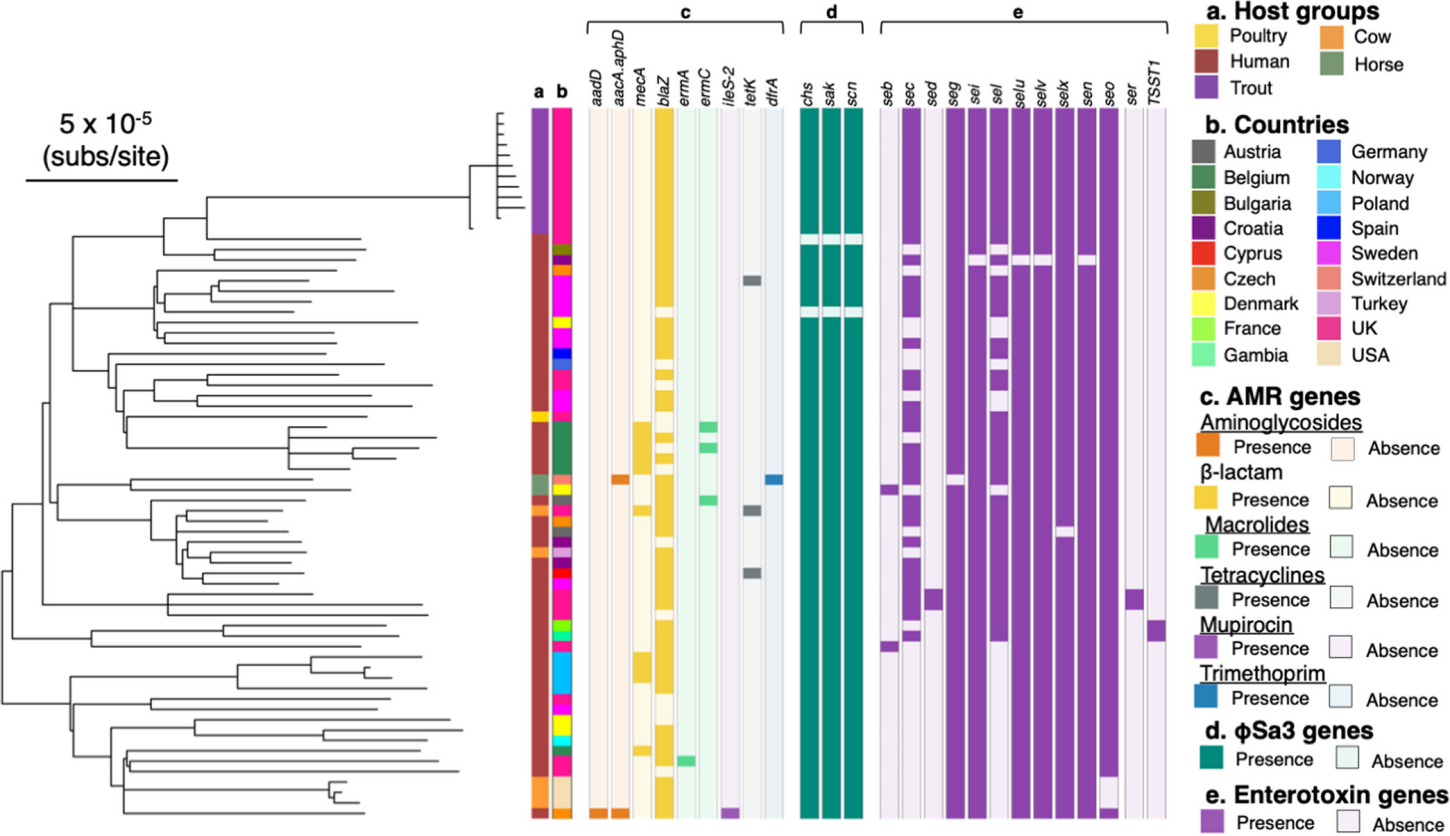
S. aureus isolates from a single rainbow trout purchased from a fish farm in London, England, fall within the diversity of CC45. Shown is a maximum likelihood phylogeny of 68 isolates of CC45 based on an LGA251 reference-mapped alignment with recombination removed and rooted using an outgroup from CC398 (with branches with a bootstrap support of <70 collapsed). Outer rings describe the host groups (a) and countries (b) from which isolates were sampled and the presence and absence of AMR genes (c), φSa3 prophage functional genes (d), and enterotoxin genes (e).

To place the farmed rainbow trout isolates within the known diversity of CC45, we assembled the genomes from publicly available sequencing data from a set of CC45 isolates (see Table S1 in the supplemental material). CC45 was mostly associated with human hosts (71%) but was also identified in nonhuman hosts. The phylogeny of CC45 shows that all of the samples that we collected from the farmed trout fall in a single clade, with a human S. aureus isolate from the United Kingdom being the closest relative (Fig. 2a and b). The mean SNP distance between this closest relative and the rainbow trout clade is 165 SNPs. Assuming the same clock rate as the one mentioned above, we estimate that the fish clade diverged from the human isolate around 1995. We identified genes carried on a φSa3 prophage that is known to be associated with human immune evasion in most isolates in our sample of CC45, including those from the farmed rainbow trout (Fig. 2d). This suggests that this CC is adapted to the human host and that its presence in other species is a consequence of recent spillover.

CC45 is, in general, methicillin sensitive (41), and only 2/68 of the isolates in our sample carry the gene associated with methicillin resistance (*mecA*). Genes associated with resistance to other antibiotics are also rare in this CC. Consistent with the low levels of resistance in this clade, the isolates from the farmed trout were found to be methicillin sensitive and to carry no resistance genes except for the *blaZ* gene that confers resistance to benzylpenicillin (Fig. 2c). Phenotypic resistance was confirmed by selecting two representative isolates, both of which matched the genotypic data (Table S2).

We investigated the potential of CC45 isolates to cause staphylococcal food poisoning by checking for the presence of staphylococcal enterotoxins. The isolates that we sampled from the farmed fish carry nine enterotoxin-encoding genes, which are common in CC45.

### Absence of S. aureus in wild populations of brown trout.

**FIG 3:**
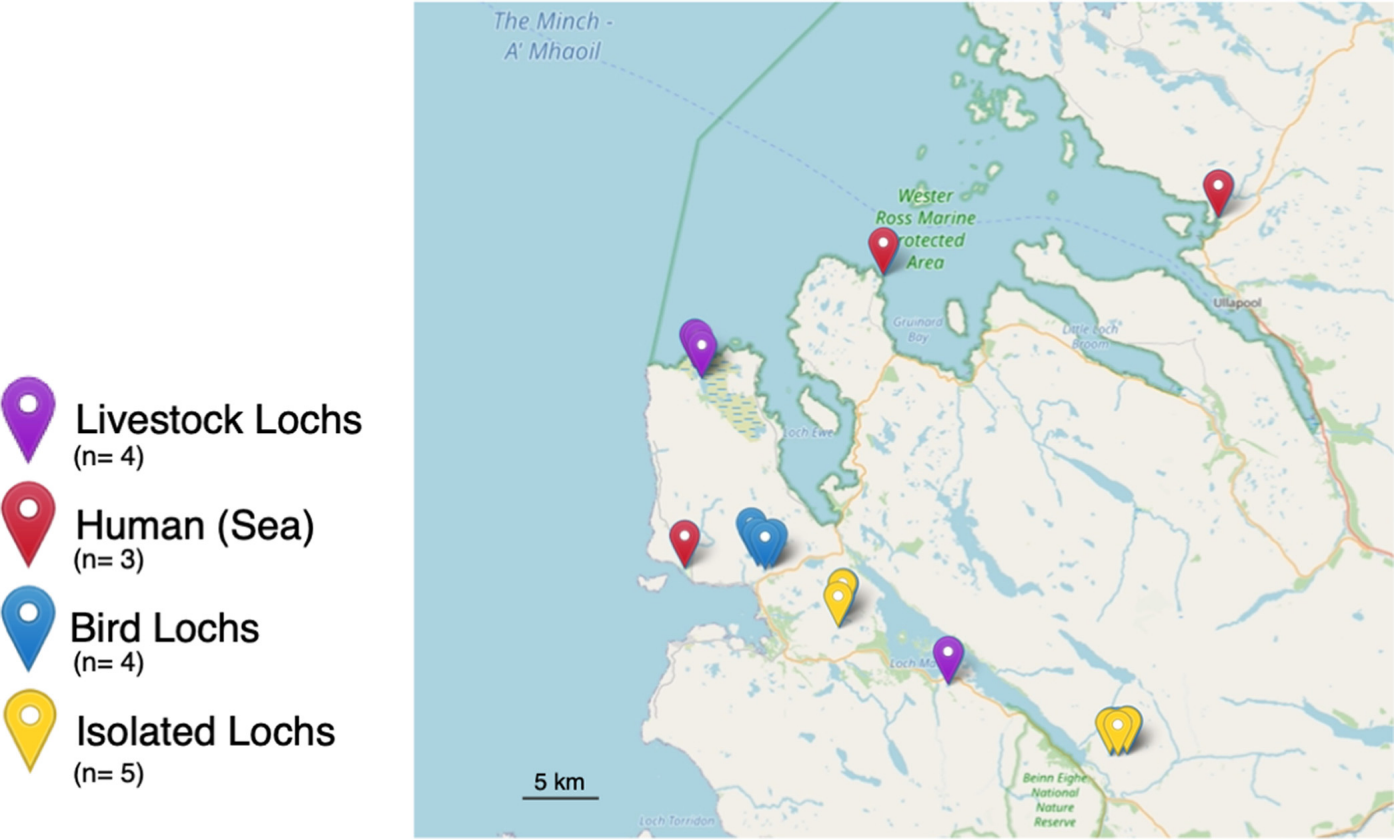
Locations of all of the sampling sites in Wester Ross, Scotland. The sites include bird, isolated, and livestock lochs and sea. The map was constructed using the R (76) Leaflet library (v2.0.4.1).

We sampled fish from four habitat types that are likely to show variation in spillover exposure. These are habitats with exposure to (i) human, (ii) livestock, and (iii) avian hosts as well as (iv) very isolated sampling sites (Fig. 3). We aimed to sample 30 fish from each of our habitats and to collect a total of 120 fish (see Materials and Methods). While our eventual sample size was 123, we were unable to reach the desired number of trout samples from the sea (exposure to human populations). We sampled 123 brown trout from 16 sites from 4 habitats (between 1 and 11 fish from each loch). We collected water and sediment samples from 23 sites from 4 habitats and 3 additional sites not categorized into any of the habitats (Tables S3 to S5). All researchers carrying out fishing and sampling were swabbed and found to be negative for S. aureus.

We did not detect any positive samples in any of the 123 wild brown trout or their environment (Table 2). Overall, these results suggest that S. aureus is either absent or present at a very low prevalence in Scottish lochs. We also conducted a thorough search for published bacterial metagenomic sequencing studies of wild fish, but unfortunately, we did not find any studies meeting these criteria.

**TABLE 2:** Prevalence of S. aureus in the environment and from brown trout in different habitats

Habitat	No. of sampled lochs or sites	No. of environmental samples	No. of positive environmental samples	No. of trout samples	No. of positive isolates	Prevalence in trout (95% CI)
All	16	32	0	123	0	0 (0−0.03)
Bird lochs	4	8	0	42	0	0 (0–0.08)
Isolated lochs	5	10	0	48	0	0 (0–0.07)
Livestock lochs	4	8	0	30	0	0 (0–0.12)
Humans (sea)	3	6	0	3	0	0 (0–0.71)

## DISCUSSION

Our study did not detect S. aureus in wild populations of brown trout or in the Scottish lochs that they inhabit. All sites with different (although all relatively low) levels of exposure to species that are known hosts of S. aureus, including livestock, humans, and birds, were negative for S. aureus. This suggests the absence or a very low prevalence of S. aureus in these populations. Our results are consistent with those of another study testing for the presence of S. aureus from different species of 168 wild fish caught by trawling and rod fishing in sea populations from Japan, where no S. aureus was detected (42). Overall, these results argue that the presence of S. aureus in farmed fish and fish products is not due to the adaptation of S. aureus to wild fish populations.

Previous studies have documented the presence of S. aureus, including MRSA, on fish farms and in fish products (32, 33). We detected S. aureus in both the internal and external organs of a single farmed fish using aseptic techniques, suggesting that its presence is not due to contamination via handling. S. aureus could be adapted to and persist within the fish host. However, the S. aureus genomic diversity within the single fish is consistent with a date of acquisition (i.e., approximately 1.5 years) that is likely older than the life span of the fish (the fish weight was 300 g, which is consistent with an age of <8 months [43]). Given the lack of clustering by anatomic location, it is more likely that the fish acquired S. aureus through the passive filtration of its external environment. However, within-fish persistence cannot be excluded due to the error bars overlapping the age of the fish. Previous studies suggested that MRSA can also survive for extended periods of time in seawater, river water, and marine freshwater (44, 45).

S. aureus may be adapted to the conditions of aquaculture. We detected the *blaZ* gene in the fish farm isolates. The production of β-lactamase (encoded by the *blaZ* gene) renders S. aureus resistant to benzylpenicillin, phenoxymethylpenicillin, ampicillin, amoxicillin, piperacillin, and ticarcillin (46). Amoxicillin and ampicillin are commonly used in aquaculture worldwide (47, 48). According to the *UK Veterinary Antibiotic Resistance and Sales Surveillance Report*, oxytetracycline, oxolinic acid, florfenicol, and amoxicillin were used in trout farming between 2017 and 2019 (49). Therefore, the *blaZ* gene could provide S. aureus with a selective advantage over other nonresistant bacteria within this environment. Other bacteria, including Pseudomonas species, commonly found in fish have been previously associated with resistance to different antibiotics on fish farms (50). Therefore, it is crucial to investigate the transfer of antimicrobial resistance (AMR) genes among and between bacterial species in fish farms. Our results show that the fish farm rainbow trout isolates are part of CC45, which is associated with both nasal colonization and bloodstream infections in humans (41). The fish farm isolates nest within a more diverse clade of human isolates and contain a φSa3 prophage, which carries a human immune evasion cluster (21). Our results therefore suggest that the presence of S. aureus in this aquaculture environment is due to recent spillover from human populations. This is consistent with the results of another study that showed the presence of a CC398 MSSA isolate from a gilthead seabream (35), which most likely originated from a human-associated lineage of CC398 (22, 36, 37). S. aureus isolates have been recovered from municipal (51–54), hospital (55), and agricultural (56) wastewaters/sewage, representing potential sources of human environmental contamination.

We detected nine enterotoxin genes in all of our farmed fish isolates. Staphylococcal enterotoxins have also been detected in other studies of S. aureus in fish and fishery products (34, 57). Staphylococcal food poisoning is caused by the ingestion of any of the 27 characterized staphylococcal enterotoxins (27, 28). The toxins can have neurotic or superantigenic activity, which results in vomiting or fever, respectively (57). The staphylococcal enterotoxins have a high tolerance to denaturing conditions such as low pH or heat, and even when ingested in small quantities, they can be toxic to humans (58). This suggests that fish from aquaculture could constitute a risk of food poisoning (28, 59).

This study also optimized a sampling approach using farmed fish to calibrate an S. aureus isolation protocol. Previous techniques for S. aureus isolation relied on the fish being euthanized, whereas this approach allowed fish to be returned to their environment after sampling (32, 59, 60).

The absence of S. aureus in wild fish populations, combined with whole-genome sequencing of strains from a farmed fish, suggests that the presence of S. aureus in fish is the result of spillover from source populations. However, it is important to acknowledge the limitations of our study. First, our sampling of wild fish was limited to a specific geographic location. Additional sampling of different regions and habitats and other species, including collecting fish microbiome data, will confirm whether our results apply more generally. Second, our analyses of farmed fish were restricted to one sample that we collected for calibration. Therefore, further research using wider whole-genome sequencing of S. aureus from aquaculture, including the environment and fish farm workers, is needed to determine the origin and mechanisms of the persistence of S. aureus in this environment. The consumption of fish and seafood is predicted to increase by 27% by 2030, and much of this increase will be supplied by growth in the aquaculture sector (61). Aquaculture is known to introduce and amplify new pathogens (62), and it relies heavily on antibiotics to combat infectious diseases (30, 63, 64). While our study found no evidence of the adaptation of S. aureus to fish, growth in the size and density of fish farms could create more opportunities for S. aureus to adapt to aquaculture and fish and could also promote the evolution and spread of strains that are resistant to antibiotics. This would have potential impacts on both food security and human health.

## MATERIALS AND METHODS

### Calibration of S. aureus isolation from fish.

To optimize our method of isolation of S. aureus from fish, a pilot experiment was carried out on a single rainbow trout (Oncorhynchus mykiss) purchased from a fish farm in London, England. The selection of sampling sites was based on the aim to include locations that are more likely to be exposed to environmental contamination as well as internal tissues that are less susceptible to contamination. Wearing gloves, we swabbed the mouth, vent, gill, and skin with charcoal swabs (Medical Wire Transwabs) and then inoculated the samples into 10 mL of 6% NaCl Staphylococcus-selective medium (A&E Laboratories). The rainbow trout was euthanized via a blow to the back of the head with a sterilized priest. We dissected and swabbed the intestine, gill, skin, and heart; transferred each organ to a sealed processing bag containing 10 mL 6% NaCl Staphylococcus-selective medium; and homogenized the organs with a stomacher (Stomacher80 laboratory system; Seward Ltd., UK). The intestine was removed, and dissection scissors were used to open it up longitudinally. The intestinal epithelium was sampled along with the gut contents. We took nasal swabs from both researchers carrying out sample processing to control for potential contamination and processed them in the same way as described above for the fish swabs. We incubated all samples at 37°C in universal tubes for 24 h. After incubation, we plated 10 to 100 μL of each culture (depending on the culture cloudiness) onto Brilliance Staph 24 agar plates (Oxoid, UK) and incubated the culture at 37°C for 24 h. For each positive plate, we confirmed the presence of S. aureus by selecting three colonies for *femB* PCR (see the PCR protocol below).

### PCR to amplify *femB*.

To confirm the presence of S. aureus, we used colony PCR with *femB* primers. The primers were FemB1 (5′-CAT GGT TAC GAG CAT CAT GG) and FemB2 (5′-AAC GCC AGA AGC AAG GTT TA), leading to an S. aureus-specific 447-bp PCR product (65). We touched a pipette tip onto a single S. aureus colony and mixed it with 20 μL of water in a PCR tube. We boiled the samples for 5 min at 95°C. Each sample contained 2 μL of a boiled cell solution and 18 μL of the PCR master mix with MyTaq DNA polymerase (Bioline). The PCR cycling conditions were 95°C 5 min; 30 cycles of 95°C for 15 s, 58°C for 10 s, and 72°C for 30 s; and 72°C for 10 min. We added 2.5 μL Sybr Safe DNA gel stain (Thermo Fisher, UK) to 100 μL of an agarose solution, which contained 1% (wt/vol) agarose dissolved by heating in 1× TBE (Tris-borate-EDTA) buffer. We loaded 15 μL of the PCR mixture into each well, with a 100-bp 5-μL 5 HyperLadder (Bioline) to confirm the product size. Electrophoresis was performed in a Sigma-Aldrich electrophoresis tank with 1× TBE at 100 V for 40 min. Electrophoresed gels were visualized under blue light, and their images were obtained with the GelDoc XR system imager (Bio-Rad).

### Sequencing.

For all confirmed and suspected S. aureus-positive samples from the farmed fish, two colonies were selected for each sampling method (swabbing and tissue sampling) per positive sampling site (gill, intestine, and skin). Genomic DNA was extracted from cultures grown overnight in tryptic soy broth (TSB) at 37°C with shaking at 200 rpm using the MasterPure Gram-positive DNA purification kit (Cambio, UK). Illumina library preparation and Hi-Seq sequencing were carried out as described previously (65).

### Genome assembly and multilocus sequence typing (MLST).

Published genome sequence data from isolates of CC45 were downloaded from the European Nucleotide Archive (ENA) and subsampled to represent host and geographic diversity (see Table S1 in the supplemental material). Sequence data from both newly sequenced and publicly available CC45 isolates were assembled using Spades v3.12.0 (66). Adapters and low-quality reads were removed with Cutadapt v1.16 (67) and Sickle v1.33 (68) and screened for contamination using FastQ Screen v0.12.0 (69). We identified optimal k-mers based on the average read lengths for each genome. All *de novo* assemblies were evaluated using QUAST v5.0.1 (70), and reads were mapped back to *de novo* assemblies to investigate polymorphisms (indicative of mixed cultures) using Bowtie2 v1.2.2 (71). All assemblies were of good quality (i.e., *N*_50_ of <10,000, contigs of <1 kb contributing to >15% of the total assembly length, total assembly length outside the median sequence length ± 1 standard deviation, or >1,500 polymorphic sites).

### Phylogenetic analyses and genome annotation.

Reference-mapped assemblies were generated using Bowtie2 v1.2.2, using the LGA251 reference genome (GenBank assembly accession no. GCA_000237265.1) (71). All genomes had an average coverage of <50× or >10% missing sites. Recombination was identified in the reference-mapped alignment using Gubbins v2.3.1, and recombinant sites were masked from phylogenetic analyses (72). Sites that had either recombination detected or missing data were excluded from phylogenetic analyses. Phylogenetic reconstruction was carried out for the reference-mapped alignment with RAxML (v8.2.4) using the *GTR*+Γ model and 1,000 bootstraps (73) and rooted using an isolate from CC398 (NCBI Sequence Read Archive accession no. SRR445234). To investigate the diversity among closely related isolates, we examined the reference-mapped alignment to identify single nucleotide polymorphisms (SNPs). The alignment after recombination stripping was uploaded to Geneious 2020.0.4 (https://www.geneious.com), and all regions that were within 100 bp of large regions containing missing data and regions containing SNP clusters of >5 SNPs located within 50 bp were removed. Next, the SNPs were extracted, and an MSTree V2 tree was constructed using GrapeTree (74). Additionally, to estimate the age of the most recent common ancestor of S. aureus in the farmed fish, we used *BEAST* v1.10 with an *HKY+Γ* model and a strict molecular clock, using substitution rates from published data sets (uniform prior distribution, where the initial value was set to 1.66 × 10^−6^ nucleotides/site/year, the upper value was set to 2.9 × 10^−6^ nucleotides/site/year, and the lower value was set to 0.64 × 10^−6^ nucleotides/site/year), removing the appropriate burn-in and run for approximately 100,000,000 generations (25).

We identified antibiotic resistance genes using the Pathogenwatch AMR prediction module (Oxford Big Data Institute), which uses BLASTn (75) with a cutoff of 75% coverage and an 80 to 90% identity threshold (depending on the gene) against an S. aureus antimicrobial resistance database. The presence of φSa3 prophages was established by searching for genes in the human immune evasion gene cluster using BLASTn (75) with a cutoff of 90% coverage and a 90% identity threshold. The query human immune evasion genes were extracted from a reference genome (GenBank assembly accession no. GCA_900324385.1). The presence of enterotoxins was investigated by searching for relevant proteins from a database compiled by Merda et al. using tBLASTn, with a cutoff of 80% coverage and an 80% identity threshold (76).

### Sample size and statistical analysis.

To guide sample size design, we calculated the probability of detecting one or more positive samples with various levels of prevalence using the pbinom function within the R software package (77). We show that a sample size of 30 fish gives us a 95% probability of detecting a positive sample if the true prevalence is around 10% (see Fig. S1 in the supplemental material). The prevalence of S. aureus in fish varies but is typically >10% (26, 33). Therefore, we chose to sample 30 fish from each of our four habitats. In total, a sample size of 120 fish gives a 95% probability of detecting a positive sample if the true prevalence at all sites is around 3%. We calculated the 95% confidence intervals shown in Table 2 based on our observed prevalence using the binom.test function in R (77).

### Fish and environmental sampling.

All sampling was carried out in July 2019. Brown trout were captured by fly fishing and transferred, along with fresh loch water/seawater, to a sterilized bucket. We swabbed the vents and gills while the trout was alive, with operators wearing sterile gloves to prevent cross-contamination. Fish larger than 250 mm were released (a condition of the landowners). We euthanized fish smaller than 250 mm via a blow to the back of the head with a sterilized priest, placed them into a specimen bag, and stored them in an icebox until they were transferred to a −80°C freezer for long-term storage. The sites were selected based on their isolation from (isolated lochs) or their proximity to (<1 km) human-frequented sites, including camping sites (human lochs); livestock grazing sites (livestock lochs); and bird nesting sites (bird lochs). All habitat types, except for bird lochs, were represented by either two or three site clusters >21 km apart.

To test whether S. aureus was present in the environment, we collected water and sediment samples from 25 sites, including all sites where we sampled trout. Sampling was carried out with operators wearing sterile gloves to prevent cross-contamination. We took water (3.5 L) from an ~5- to 10-cm depth using sterile 1,000-mL and 500-mL containers. We collected sediment (approximately 50 g per location) in sterile containers using a sterilized plastic scoop from littoral areas that were (i) >150 mm below the water surface and (ii) undisturbed. We kept both water and sediment samples chilled at 4°C until analysis. Samples were processed immediately upon arrival at the laboratory. We took nasal swabs from both researchers carrying out sample processing to control for potential contamination.

### S. aureus isolation from environmental samples.

To isolate S. aureus from water, we filtered three aliquots of 1,000 mL per water sample through 0.45-μm 47-mm white gridded mixed ester cellulose membranes (Merck, USA). The membranes were placed onto 55-mm plates containing Baird-Parker enrichment agar with potassium tellurite and incubated at 37°C for 48 h. To isolate S. aureus from sediment, we transferred three 10-g (wet weight) sediment samples (per location) to sterile 50-mL centrifuge tubes. We added 25 mL of 2-fold-concentrated Baird-Parker enrichment medium with potassium tellurite to each sample. After vortexing for approximately 45 s, the supernatant from each sample was transferred to a new sterile tube and incubated until a black precipitate formed (up to 7 days). After this, the samples were vortexed for 30 s, and 100 μL was plated onto 100-mm plates containing Baird-Parker enrichment agar with potassium tellurite and incubated at 37°C for 48 h. For both water and sediment samples, after incubation, we transferred three presumptive S. aureus colonies (black colonies) onto fresh Brilliance Staph 24 plates (Oxoid, UK) and incubated them at 37°C for 24 h. For each potentially positive plate, we confirmed the presence of S. aureus by selecting three colonies for *femB* PCR (see the PCR protocol above).

### Antibiotic susceptibility testing.

Antibiotic susceptibility testing was carried out with the Vitek 2 system (AES software; bioMérieux, Marcy l’Etoile, France) according to the manufacturer’s instructions. The isolates were plated onto Columbia blood agar (Oxoid Deutschland) and incubated at 37°C for 18 to 24 h. One colony was picked with a sterile swab and mixed in saline by vortexing. The optical density at 600 nm (OD_600_) was measured and adjusted to 0.5 to 0.63. The inoculum was prepared by transferring 280 μL to 3 mL saline. Next, each sample was loaded into a Vitek 2 AST-GP80 card. Susceptibility cards were interpreted according to Clinical and Laboratory Standards Institute (CLSI) breakpoints (78).

### S. aureus detection in the wild-fish microbiome.

To explore the potential presence of S. aureus in wild fish, we utilized the MGnify EBML-EBI catalogue (79) to search for all published metagenomic sequencing studies associated with the fish biome. We narrowed our focus to studies on wild fish and excluded 16S rRNA data due to their unreliable species-level classifications (previously, only 6.9 to 18.9% of gene amplicons were correctly assigned to a species [80]).

### Data availability.

The raw sequence reads generated from Illumina high-throughput sequencing have been deposited in the NCBI Sequence Read Archive database under accession no. ERR4188682, ERR4188685, ERR4188688, ERR4188691, ERR4188694, ERR4188697, ERR4188700, ERR4188703, ERR4188705, ERR4188708, ERR4188711, and ERR4188714, with BioProject study accession no. PRJEB21015 (Table S6).
