# Supplementary material for: Absence of Staphylococcus aureus in Wild Populations of Fish Supports a Spillover Hypothesis
Source: Microbiol Spectr. 2023 Jun 21;11(4):e04858-22. doi: 10.1128/spectrum.04858-22 (PMC10434045; doi:10.1128/spectrum.04858-22)
Supplement: Supplemental file 1 — Table S1. Download spectrum.04858-22-s0007.pdf, PDF file, 0.06 MB [file spectrum.04858-22-s0007.pdf]

Table S1: Metadata for all CC45 *S. aureus* used in the phylogenetic reconstruction analysis.

Strain	*County*	*Host*	*ST*	*CC*	*ERR number*
09.4504.Z	United Kingdom	Human	45	45	ERR084607
4238_1_1	Belgium	Human	45	45	ERR033239
4238_1_10	Belgium	Human	45	45	ERR033240
4238_1_7	Belgium	Human	45	45	ERR033248
4238_1_8	Belgium	Human	45	45	ERR033249
4330_8_1	Austria	Human	45	45	ERR033304
4340_5_1	Bulgaria	Human	45	45	ERR033330
4340_6_1	Belgium	Human	45	45	ERR033343
4350_1_6	Czech	Human	45	45	ERR033364
4386_1_11	Croatia	Human	45	45	ERR033410
4386_1_2	Croatia	Human	45	45	ERR033412
4386_1_3	Cyprus	Human	45	45	ERR033413
4386_1_5	Croatia	Human	45	45	ERR033415
4386_7_4	Germany	Human	45	45	ERR033479
4395_3_11	Spain	Human	45	45	ERR033526
4414_2_10	Poland	Human	45	45	ERR033551
4414_2_6	Poland	Human	45	45	ERR033558
4414_3_10	Sweden	Human	45	45	ERR033564
4414_3_11	Sweden	Human	45	45	ERR033565
4414_3_12	Sweden	Human	45	45	ERR033566
4414_3_3	Sweden	Human	45	45	ERR033568
4414_5_10	Sweden	Human	45	45	ERR033577
4414_5_4	Sweden	Human	45	45	ERR033582
4414_5_6	Sweden	Human	45	45	ERR033584
4414_6_3	Sweden	Human	45	45	ERR033594
4414_6_6	Sweden	Human	45	45	ERR033597
4430_1_1	Denmark	Human	45	45	ERR033628
4465_5_4	Norway	Human	45	45	ERR033712
6133_3_5	Poland	Human	45	45	ERR038686
6133_3_8	Poland	Human	45	45	ERR038689
6236_1_4	Austria	Human	45	45	ERR039391
A-69	Turkey	Cow	45	45	ERR387222
B302368	United Kingdom	Wild Bird	45	45	ERR2505672
CSRJAE39	Switzerland	Horse	45	45	ERR234859
CTH14	USA	Cow	45	45	ERR2505706
CTH160	USA	Cow	45	45	ERR2505707
CTH211	USA	Cow	45	45	ERR2505703
Z_22	Denmark	Horse	45	45	ERR2505688
4414_5_8	Sweden	Human	46	45	ERR033586
4415_2_8	United Kingdom	Human	46	45	ERR033612

ASASM145	United Kingdom	Human	47	45	ERR109591
ASASM300	United Kingdom	Human	54	45	ERR114905
ASASM325	United Kingdom	Human	508	45	ERR114924
SA21	Gambia	Human	508	45	ERR1213778
4386_2_1	Denmark	Human	682	45	ERR033421
4350_1_7	Czech	Human	2860	45	ERR033365
4386_2_6	Denmark	Human	2863	45	ERR033429
6133_1_12	France	Human	2876	45	ERR038669
ASASM17	United Kingdom	Human	2902	45	ERR109499
ASASM158	United Kingdom	Human	2903	45	ERR109571
ASASM356	United Kingdom	Human	2904	45	ERR109683
ASASM171	United Kingdom	Human	2927	45	ERR109616
ASASM426	United Kingdom	Human	2940	45	ERR172064
4330_8_4	Belgium	Human	3299	45	ERR033310
45-164	United Kingdom	Cow	3613	45	ERR294319
4350_1_4	Czech	Human	4670	45	ERR033362
