# Supplementary material for: Absence of Staphylococcus aureus in Wild Populations of Fish Supports a Spillover Hypothesis
Source: Microbiol Spectr. 2023 Jun 21;11(4):e04858-22. doi: 10.1128/spectrum.04858-22 (PMC10434045; doi:10.1128/spectrum.04858-22)
Supplement: Supplemental file 2 — Table S2. Download spectrum.04858-22-s0001.pdf, PDF file, 0.06 MB [file spectrum.04858-22-s0001.pdf]

Table S2: MIC values obtained using Vitek2 for two rainbow trout isolates from fish farm in London.

Antibiotic	*BTGL0319001*	*BTIL0319003*
Cefoxitin Screen	NEG	NEG
Benzylpenicillin	>=0.5	>=0.5
Amoxicillin/ Clavulanic acid	<=2	<=2
Oxacillin	<=0.25	<=0.25
Cefalotin	<=2	<=2
Cefovecin	1	1
Ceftiofur	1	1
Gentamicin	<=0.5	<=0.5
Kanamycin	<=4	<=4
Neomycin	<=2	<=2
Enrofloxacin	<=0.5	<=0.5
Marbofloxacin	<=0.5	<=0.5
Pradofloxacin	<=0.12	<=0.12
Inducible Clindamycin Resistance (ICR)	NEG	NEG
Erythromycin	<=0.25	<=0.25
Clindamycin	0.25	0.25
Doxycycline	<=0.5	<=0.5
Tetracycline	<=1	<=1
Nitrofurantoin	32	32
Chloramphenicol	8	<=4
Trimethoprim / Sulfamethoxazole	<=10	<=10
