# Supplementary material for: Absence of Staphylococcus aureus in Wild Populations of Fish Supports a Spillover Hypothesis
Source: Microbiol Spectr. 2023 Jun 21;11(4):e04858-22. doi: 10.1128/spectrum.04858-22 (PMC10434045; doi:10.1128/spectrum.04858-22)
Supplement: Supplemental file 3 — Table S3. Download spectrum.04858-22-s0002.pdf, PDF file, 0.05 MB [file spectrum.04858-22-s0002.pdf]

Table S3: Sampled lochs with sizes, coordinates.

Sampling site	*Location*	*Loch code*	*Perimeter (km)*	*Area (ha)*	*Altitude (m)*	*Habitat*	*Sampling date*
Loch a'Gharbh Doire	N57°44'52" W5°41'51"	LGD	1.69	10.98	82	Bird	13 JUL 2019
Loch Coire na h-Airigh	N57°44'29" W5°41'25"	LCA	0.78	2.23	80	Bird	13 JUL 2019
Loch Feur	N57°44'21" W5°40'57"	LFE	1.16	4.55	75	Bird	13 JUL 2019
Loch nam Breac	N57°44'27" W5°40'30"	LNB	1.02	4.95	77	Bird	13 JUL 2019
Greylag Loch	N57°54'40" W5°33'36"	GRB	0.97	4.83	21	Bird	14 JUL 2019
Loch Orchid	N57°37'47" W5°16'55"	GLO	0.44	0.87	374	Isolated	12 JUL 2019
Golden Loch A	N57°37'46" W5°17'56"	LFO	0.63	1.61	420	Isolated	12 JUL 2019
Golden Loch B	N57°37'44" W5°17'29"	LFI	0.75	1	400	Isolated	12 JUL 2019
Loch Dubh Dughaill	N57°42'18" W5°36'02"	LDD	0.73	1.66	293	Isolated	15 JUL 2019
Loch na Feithe Mugaig	N57°42'42" W5°35'46"	LFM	3.65	16.27	306	Isolated	15 JUL 2019
Loch nan Buainichean	N57°42'01" W5°36'14"	LB	2.1	11.13	204	Isolated	15 JUL 2019
Loch an Draing	N57°51'08" W5°45'10"	LDR	3.16	38	41	Livestock	11 JUL 2019

Loch nan Eun	N57°51'16" W5°45'19"	LEV	2.15	16.46	40	Livestock	11 JUL 2019
Loch na Fideil	N57°40'15" W5°28'45"	LNF	0.62	2.5	21	Livestock	17 JUL 2019
Loch Beag nan Eun	N57°51'30" W5°45'39"	LBE	0.34	0.78	42	Livestock	19 JUL 2019
Loch Bad an Scalaig	N57°41'11.12"W5°36'42.22"	LBS			114	NA	22 JUL 2019
Allt Phadraig	N57°46'44" W5°48'00"	APD				NA	19 JUL 2019
Kerrysdale (River Kerry B8056 A832)	N57°41'35.34"W5°39'27.41"	KED			24	NA	22 JUL 2019
Pollack Point	N57°54'45" W5°33'05"	SPP	NA	NA	NA	Sea	14 JUL 2019
Mellon Udrigle Bay	N57°54'12" W5°33'22"	MUB	NA	NA	NA	Sea	14 JUL 2019
River Canaird	N57°56'50" W5°10'48"	RCA	NA	NA	NA	Sea	16 JUL 2019
Sand River	N57°44'25" W5°46'19"	SR	NA	NA	NA	Sea	21 JUL 2019
Flowerdale Estuary	N57°42'44" W5°40'43"	FES	NA	NA	NA	Sea	20 JUL 2019
Sand Beach Ocean	N57°44'11" W5°46'07"	SBO	NA	NA	NA	Sea	21 JUL 2019
