# Supplementary material for: Absence of Staphylococcus aureus in Wild Populations of Fish Supports a Spillover Hypothesis
Source: Microbiol Spectr. 2023 Jun 21;11(4):e04858-22. doi: 10.1128/spectrum.04858-22 (PMC10434045; doi:10.1128/spectrum.04858-22)
Supplement: Supplemental file 4 — Table S4. Download spectrum.04858-22-s0003.pdf, PDF file, 0.09 MB [file spectrum.04858-22-s0003.pdf]

Table S4: Metadata for all samples collected from fish in Scottish Highlands. The loch code corresponds to names in the Table S3.

Sample name	*Tissue sampled*	*Loch code*	*Habitat*	*Host*	*Date of sampling*
BTG_LNB0719_001	Gills	LNB	Bird Lochs	Brown Trout	9 JUL 2019
BTG_LNB0719_002	Gills	LNB	Bird Lochs	Brown Trout	9 JUL 2019
BTG_LNB0719_003	Gills	LNB	Bird Lochs	Brown Trout	9 JUL 2019
BTG_LNB0719_004	Gills	LNB	Bird Lochs	Brown Trout	9 JUL 2019
BTG_LNB0719_005	Gills	LNB	Bird Lochs	Brown Trout	9 JUL 2019
BTG_LNB0719_006	Gills	LNB	Bird Lochs	Brown Trout	9 JUL 2019
BTG_LNB0719_007	Gills	LNB	Bird Lochs	Brown Trout	9 JUL 2019
BTG_LNB0719_008	Gills	LNB	Bird Lochs	Brown Trout	9 JUL 2019
BTG_LNB0719_009	Gills	LNB	Bird Lochs	Brown Trout	9 JUL 2019
BTG_LNB0719_010	Gills	LNB	Bird Lochs	Brown Trout	9 JUL 2019
BTV_LNB0719_001	Vent	LNB	Bird Lochs	Brown Trout	9 JUL 2019
BTV_LNB0719_002	Vent	LNB	Bird Lochs	Brown Trout	9 JUL 2019
BTV_LNB0719_003	Vent	LNB	Bird Lochs	Brown Trout	9 JUL 2019
BTV_LNB0719_004	Vent	LNB	Bird Lochs	Brown Trout	9 JUL 2019
BTV_LNB0719_005	Vent	LNB	Bird Lochs	Brown Trout	9 JUL 2019
BTV_LNB0719_006	Vent	LNB	Bird Lochs	Brown Trout	9 JUL 2019
BTV_LNB0719_007	Vent	LNB	Bird Lochs	Brown Trout	9 JUL 2019
BTV_LNB0719_008	Vent	LNB	Bird Lochs	Brown Trout	9 JUL 2019
BTV_LNB0719_009	Vent	LNB	Bird Lochs	Brown Trout	9 JUL 2019
BTV_LNB0719_010	Vent	LNB	Bird Lochs	Brown Trout	9 JUL 2019
BTG_LGD0719_001	Gills	LGD	Bird Lochs	Brown Trout	10 JUL 2019
BTG_LGD0719_002	Gills	LGD	Bird Lochs	Brown Trout	10 JUL 2019
BTG_LGD0719_003	Gills	LGD	Bird Lochs	Brown Trout	10 JUL 2019
BTG_LGD0719_004	Gills	LGD	Bird Lochs	Brown Trout	10 JUL 2019
BTG_LGD0719_005	Gills	LGD	Bird Lochs	Brown Trout	10 JUL 2019
BTG_LGD0719_006	Gills	LGD	Bird Lochs	Brown Trout	10 JUL 2019
BTG_LGD0719_007	Gills	LGD	Bird Lochs	Brown Trout	10 JUL 2019
BTG_LGD0719_008	Gills	LGD	Bird Lochs	Brown Trout	10 JUL 2019
BTG_LGD0719_009	Gills	LGD	Bird Lochs	Brown Trout	10 JUL 2019
BTG_LGD0719_010	Gills	LGD	Bird Lochs	Brown Trout	10 JUL 2019
BTG_LGD0719_011	Gills	LGD	Bird Lochs	Brown Trout	10 JUL 2019
BTV_LGD0719_001	Vent	LGD	Bird Lochs	Brown Trout	10 JUL 2019
BTV_LGD0719_002	Vent	LGD	Bird Lochs	Brown Trout	10 JUL 2019
BTV_LGD0719_003	Vent	LGD	Bird Lochs	Brown Trout	10 JUL 2019
BTV_LGD0719_004	Vent	LGD	Bird Lochs	Brown Trout	10 JUL 2019
BTV_LGD0719_005	Vent	LGD	Bird Lochs	Brown Trout	10 JUL 2019
BTV_LGD0719_006	Vent	LGD	Bird Lochs	Brown Trout	10 JUL 2019
BTV_LGD0719_007	Vent	LGD	Bird Lochs	Brown Trout	10 JUL 2019
BTV_LGD0719_008	Vent	LGD	Bird Lochs	Brown Trout	10 JUL 2019
BTV_LGD0719_009	Vent	LGD	Bird Lochs	Brown Trout	10 JUL 2019
BTV_LGD0719_010	Vent	LGD	Bird Lochs	Brown Trout	10 JUL 2019
BTV_LGD0719_011	Vent	LGD	Bird Lochs	Brown Trout	10 JUL 2019
BTG_LCA0719_001	Gills	LCA	Bird Lochs	Brown Trout	13 JUL 2019
BTG_LCA0719_002	Gills	LCA	Bird Lochs	Brown Trout	13 JUL 2019
BTG_LCA0719_003	Gills	LCA	Bird Lochs	Brown Trout	13 JUL 2019
BTG_LCA0719_004	Gills	LCA	Bird Lochs	Brown Trout	13 JUL 2019
BTG_LCA0719_005	Gills	LCA	Bird Lochs	Brown Trout	13 JUL 2019
BTG_LCA0719_006	Gills	LCA	Bird Lochs	Brown Trout	13 JUL 2019
BTG_LCA0719_007	Gills	LCA	Bird Lochs	Brown Trout	13 JUL 2019
BTG_LCA0719_008	Gills	LCA	Bird Lochs	Brown Trout	13 JUL 2019
BTG_LCA0719_009	Gills	LCA	Bird Lochs	Brown Trout	13 JUL 2019
BTG_LCA0719_010	Gills	LCA	Bird Lochs	Brown Trout	13 JUL 2019
BTG_LCA0719_011	Gills	LCA	Bird Lochs	Brown Trout	13 JUL 2019
BTV_LCA0719_001	Vent	LCA	Bird Lochs	Brown Trout	13 JUL 2019
BTV_LCA0719_002	Vent	LCA	Bird Lochs	Brown Trout	13 JUL 2019

BTV_LCA0719_003	Vent	LCA	Bird Lochs	Brown Trout	13 JUL 2019
BTV_LCA0719_004	Vent	LCA	Bird Lochs	Brown Trout	13 JUL 2019
BTV_LCA0719_005	Vent	LCA	Bird Lochs	Brown Trout	13 JUL 2019
BTV_LCA0719_006	Vent	LCA	Bird Lochs	Brown Trout	13 JUL 2019
BTV_LCA0719_007	Vent	LCA	Bird Lochs	Brown Trout	13 JUL 2019
BTV_LCA0719_008	Vent	LCA	Bird Lochs	Brown Trout	13 JUL 2019
BTV_LCA0719_009	Vent	LCA	Bird Lochs	Brown Trout	13 JUL 2019
BTV_LCA0719_010	Vent	LCA	Bird Lochs	Brown Trout	13 JUL 2019
BTV_LCA0719_011	Vent	LCA	Bird Lochs	Brown Trout	13 JUL 2019
BTG_LEF0719_001	Gills	LFE	Bird Lochs	Brown Trout	13 JUL 2019
BTG_LEF0719_002	Gills	LFE	Bird Lochs	Brown Trout	13 JUL 2019
BTG_LEF0719_003	Gills	LFE	Bird Lochs	Brown Trout	13 JUL 2019
BTG_LEF0719_004	Gills	LFE	Bird Lochs	Brown Trout	13 JUL 2019
BTG_LEF0719_005	Gills	LFE	Bird Lochs	Brown Trout	13 JUL 2019
BTG_LEF0719_006	Gills	LFE	Bird Lochs	Brown Trout	13 JUL 2019
BTG_LEF0719_007	Gills	LFE	Bird Lochs	Brown Trout	13 JUL 2019
BTG_LEF0719_008	Gills	LFE	Bird Lochs	Brown Trout	13 JUL 2019
BTG_LEF0719_009	Gills	LFE	Bird Lochs	Brown Trout	13 JUL 2019
BTG_LEF0719_010	Gills	LFE	Bird Lochs	Brown Trout	13 JUL 2019
BTV_LEF0719_001	Vent	LFE	Bird Lochs	Brown Trout	13 JUL 2019
BTV_LEF0719_002	Vent	LFE	Bird Lochs	Brown Trout	13 JUL 2019
BTV_LEF0719_003	Vent	LFE	Bird Lochs	Brown Trout	13 JUL 2019
BTV_LEF0719_004	Vent	LFE	Bird Lochs	Brown Trout	13 JUL 2019
BTV_LEF0719_005	Vent	LFE	Bird Lochs	Brown Trout	13 JUL 2019
BTV_LEF0719_006	Vent	LFE	Bird Lochs	Brown Trout	13 JUL 2019
BTV_LEF0719_007	Vent	LFE	Bird Lochs	Brown Trout	13 JUL 2019
BTV_LEF0719_008	Vent	LFE	Bird Lochs	Brown Trout	13 JUL 2019
BTV_LEF0719_009	Vent	LFE	Bird Lochs	Brown Trout	13 JUL 2019
BTV_LEF0719_010	Vent	LFE	Bird Lochs	Brown Trout	13 JUL 2019
BTG_LFO0719_001	Gills	LFO	Isolated Lochs	Brown Trout	12 JUL 2019
BTG_LFO0719_002	Gills	LFO	Isolated Lochs	Brown Trout	12 JUL 2019
BTG_LFO0719_003	Gills	LFO	Isolated Lochs	Brown Trout	12 JUL 2019
BTG_LFO0719_004	Gills	LFO	Isolated Lochs	Brown Trout	12 JUL 2019
BTG_LFO0719_005	Gills	LFO	Isolated Lochs	Brown Trout	12 JUL 2019
BTG_LFO0719_006	Gills	LFO	Isolated Lochs	Brown Trout	12 JUL 2019
BTG_LFO0719_007	Gills	LFO	Isolated Lochs	Brown Trout	12 JUL 2019
BTG_LFI0719_001	Gills	LFI	Isolated Lochs	Brown Trout	12 JUL 2019
BTG_LFI0719_002	Gills	LFI	Isolated Lochs	Brown Trout	12 JUL 2019
BTG_GLO0719_001	Gills	GLO	Isolated Lochs	Brown Trout	12 JUL 2019
BTG_GLO0719_002	Gills	GLO	Isolated Lochs	Brown Trout	12 JUL 2019
BTG_GLO0719_003	Gills	GLO	Isolated Lochs	Brown Trout	12 JUL 2019
BTG_GLO0719_004	Gills	GLO	Isolated Lochs	Brown Trout	12 JUL 2019
BTG_GLO0719_005	Gills	GLO	Isolated Lochs	Brown Trout	12 JUL 2019
BTG_GLO0719_006	Gills	GLO	Isolated Lochs	Brown Trout	12 JUL 2019
BTG_GLO0719_007	Gills	GLO	Isolated Lochs	Brown Trout	12 JUL 2019
BTG_GLO0719_008	Gills	GLO	Isolated Lochs	Brown Trout	12 JUL 2019
BTG_GLO0719_009	Gills	GLO	Isolated Lochs	Brown Trout	12 JUL 2019
BTG_GLO0719_010	Gills	GLO	Isolated Lochs	Brown Trout	12 JUL 2019
BTG_LEM0719_001	Gills	LFM	Isolated Lochs	Brown Trout	15 JUL 2019
BTG_LEM0719_002	Gills	LFM	Isolated Lochs	Brown Trout	15 JUL 2019
BTG_LEM0719_003	Gills	LFM	Isolated Lochs	Brown Trout	15 JUL 2019
BTG_LEM0719_004	Gills	LFM	Isolated Lochs	Brown Trout	15 JUL 2019
BTG_LEM0719_005	Gills	LFM	Isolated Lochs	Brown Trout	15 JUL 2019
BTG_LEM0719_006	Gills	LFM	Isolated Lochs	Brown Trout	15 JUL 2019
BTG_LEM0719_007	Gills	LFM	Isolated Lochs	Brown Trout	15 JUL 2019
BTG_LEM0719_008	Gills	LFM	Isolated Lochs	Brown Trout	15 JUL 2019
BTG_LEM0719_009	Gills	LFM	Isolated Lochs	Brown Trout	15 JUL 2019
BTG_LEM0719_010	Gills	LFM	Isolated Lochs	Brown Trout	15 JUL 2019
BTG_LEM0719_011	Gills	LFM	Isolated Lochs	Brown Trout	15 JUL 2019
BTG_LEM0719_012	Gills	LFM	Isolated Lochs	Brown Trout	15 JUL 2019

BTG_LEM0719_013	Gills	LFM	Isolated Lochs	Brown Trout	15 JUL 2019
BTG_LEM0719_014	Gills	LFM	Isolated Lochs	Brown Trout	15 JUL 2019
BTG_LEM0719_015	Gills	LFM	Isolated Lochs	Brown Trout	15 JUL 2019
BTG_LEM0719_016	Gills	LFM	Isolated Lochs	Brown Trout	15 JUL 2019
BTG_LEM0719_017	Gills	LFM	Isolated Lochs	Brown Trout	15 JUL 2019
BTG_LEM0719_018	Gills	LFM	Isolated Lochs	Brown Trout	15 JUL 2019
BTG_LEM0719_019	Gills	LFM	Isolated Lochs	Brown Trout	15 JUL 2019
BTG_LEM0719_020	Gills	LFM	Isolated Lochs	Brown Trout	15 JUL 2019
BTG_LEM0719_021	Gills	LFM	Isolated Lochs	Brown Trout	15 JUL 2019
BTV_LEM0719_001	Gills	LFM	Isolated Lochs	Brown Trout	15 JUL 2019
BTG_LDD0719_001	Gills	LDD	Isolated Lochs	Brown Trout	15 JUL 2019
BTG_LDD0719_002	Gills	LDD	Isolated Lochs	Brown Trout	15 JUL 2019
BTG_LDD0719_003	Gills	LDD	Isolated Lochs	Brown Trout	15 JUL 2019
BTG_LDD0719_004	Gills	LDD	Isolated Lochs	Brown Trout	15 JUL 2019
BTG_LDD0719_005	Gills	LDD	Isolated Lochs	Brown Trout	15 JUL 2019
BTG_LDD0719_006	Gills	LDD	Isolated Lochs	Brown Trout	15 JUL 2019
BTG_LDD0719_007	Gills	LDD	Isolated Lochs	Brown Trout	15 JUL 2019
BTG_LDD0719_008	Gills	LDD	Isolated Lochs	Brown Trout	15 JUL 2019
BTV_LFO0719_001	Vent	LFO	Isolated Lochs	Brown Trout	12 JUL 2019
BTV_LFO0719_002	Vent	LFO	Isolated Lochs	Brown Trout	12 JUL 2019
BTV_LFO0719_003	Vent	LFO	Isolated Lochs	Brown Trout	12 JUL 2019
BTV_LFO0719_004	Vent	LFO	Isolated Lochs	Brown Trout	12 JUL 2019
BTV_LFO0719_005	Vent	LFO	Isolated Lochs	Brown Trout	12 JUL 2019
BTV_LFO0719_006	Vent	LFO	Isolated Lochs	Brown Trout	12 JUL 2019
BTV_LFO0719_007	Vent	LFO	Isolated Lochs	Brown Trout	12 JUL 2019
BTV_LFI0719_001	Vent	LFI	Isolated Lochs	Brown Trout	12 JUL 2019
BTV_LFI0719_002	Vent	LFI	Isolated Lochs	Brown Trout	12 JUL 2019
BTV_GLO0719_001	Vent	GLO	Isolated Lochs	Brown Trout	12 JUL 2019
BTV_GLO0719_002	Vent	GLO	Isolated Lochs	Brown Trout	12 JUL 2019
BTV_GLO0719_003	Vent	GLO	Isolated Lochs	Brown Trout	12 JUL 2019
BTV_GLO0719_004	Vent	GLO	Isolated Lochs	Brown Trout	12 JUL 2019
BTV_GLO0719_005	Vent	GLO	Isolated Lochs	Brown Trout	12 JUL 2019
BTV_GLO0719_006	Vent	GLO	Isolated Lochs	Brown Trout	12 JUL 2019
BTV_GLO0719_007	Vent	GLO	Isolated Lochs	Brown Trout	12 JUL 2019
BTV_GLO0719_008	Vent	GLO	Isolated Lochs	Brown Trout	12 JUL 2019
BTV_GLO0719_009	Vent	GLO	Isolated Lochs	Brown Trout	12 JUL 2019
BTV_GLO0719_010	Vent	GLO	Isolated Lochs	Brown Trout	12 JUL 2019
BTV_LEM0719_002	Vent	LFM	Isolated Lochs	Brown Trout	15 JUL 2019
BTV_LEM0719_003	Vent	LFM	Isolated Lochs	Brown Trout	15 JUL 2019
BTV_LEM0719_004	Vent	LFM	Isolated Lochs	Brown Trout	15 JUL 2019
BTV_LEM0719_005	Vent	LFM	Isolated Lochs	Brown Trout	15 JUL 2019
BTV_LEM0719_006	Vent	LFM	Isolated Lochs	Brown Trout	15 JUL 2019
BTV_LEM0719_007	Vent	LFM	Isolated Lochs	Brown Trout	15 JUL 2019
BTV_LEM0719_008	Vent	LFM	Isolated Lochs	Brown Trout	15 JUL 2019
BTV_LEM0719_009	Vent	LFM	Isolated Lochs	Brown Trout	15 JUL 2019
BTV_LEM0719_010	Vent	LFM	Isolated Lochs	Brown Trout	15 JUL 2019
BTV_LEM0719_011	Vent	LFM	Isolated Lochs	Brown Trout	15 JUL 2019
BTV_LEM0719_012	Vent	LFM	Isolated Lochs	Brown Trout	15 JUL 2019
BTV_LEM0719_013	Vent	LFM	Isolated Lochs	Brown Trout	15 JUL 2019
BTV_LEM0719_014	Vent	LFM	Isolated Lochs	Brown Trout	15 JUL 2019
BTV_LEM0719_015	Vent	LFM	Isolated Lochs	Brown Trout	15 JUL 2019
BTV_LEM0719_016	Vent	LFM	Isolated Lochs	Brown Trout	15 JUL 2019
BTV_LEM0719_017	Vent	LFM	Isolated Lochs	Brown Trout	15 JUL 2019
BTV_LEM0719_018	Vent	LFM	Isolated Lochs	Brown Trout	15 JUL 2019
BTV_LEM0719_019	Vent	LFM	Isolated Lochs	Brown Trout	15 JUL 2019
BTV_LEM0719_020	Vent	LFM	Isolated Lochs	Brown Trout	15 JUL 2019
BTV_LEM0719_021	Vent	LFM	Isolated Lochs	Brown Trout	15 JUL 2019
BTV_LDD0719_001	Vent	LDD	Isolated Lochs	Brown Trout	15 JUL 2019
BTV_LDD0719_002	Vent	LDD	Isolated Lochs	Brown Trout	15 JUL 2019
BTV_LDD0719_003	Vent	LDD	Isolated Lochs	Brown Trout	15 JUL 2019

BTV_LDD0719_004	Vent	LDD	Isolated Lochs	Brown Trout	15 JUL 2019
BTV_LDD0719_005	Vent	LDD	Isolated Lochs	Brown Trout	15 JUL 2019
BTV_LDD0719_006	Vent	LDD	Isolated Lochs	Brown Trout	15 JUL 2019
BTV_LDD0719_007	Vent	LDD	Isolated Lochs	Brown Trout	15 JUL 2019
BTV_LDD0719_008	Vent	LDD	Isolated Lochs	Brown Trout	15 JUL 2019
BTG_LDR0719_001	Gills	LDR	Livestock Lochs	Brown Trout	11 JUL 2019
BTG_LDR0719_002	Gills	LDR	Livestock Lochs	Brown Trout	11 JUL 2019
BTV_LDR0719_001	Vent	LDR	Livestock Lochs	Brown Trout	11 JUL 2019
BTV_LDR0719_002	Vent	LDR	Livestock Lochs	Brown Trout	11 JUL 2019
BTG_LBE0719_001	Gills	LBE	Livestock Lochs	Brown Trout	19 JUN 2019
BTV_LBE0719_001	Vent	LBE	Livestock Lochs	Brown Trout	19 JUN 2019
BTG_LNF0719_001	Gills	LNF	Livestock Lochs	Brown Trout	17 JUL 2019
BTG_LNF0719_002	Gills	LNF	Livestock Lochs	Brown Trout	17 JUL 2019
BTG_LNF0719_003	Gills	LNF	Livestock Lochs	Brown Trout	17 JUL 2019
BTG_LNF0719_004	Gills	LNF	Livestock Lochs	Brown Trout	17 JUL 2019
BTG_LNF0719_005	Gills	LNF	Livestock Lochs	Brown Trout	17 JUL 2019
BTG_LNF0719_006	Gills	LNF	Livestock Lochs	Brown Trout	17 JUL 2019
BTG_LNF0719_007	Gills	LNF	Livestock Lochs	Brown Trout	17 JUL 2019
BTG_LNF0719_008	Gills	LNF	Livestock Lochs	Brown Trout	17 JUL 2019
BTG_LNF0719_009	Gills	LNF	Livestock Lochs	Brown Trout	17 JUL 2019
BTV_LNF0719_001	Vent	LNF	Livestock Lochs	Brown Trout	17 JUL 2019
BTV_LNF0719_002	Vent	LNF	Livestock Lochs	Brown Trout	17 JUL 2019
BTV_LNF0719_003	Vent	LNF	Livestock Lochs	Brown Trout	17 JUL 2019
BTV_LNF0719_004	Vent	LNF	Livestock Lochs	Brown Trout	17 JUL 2019
BTV_LNF0719_005	Vent	LNF	Livestock Lochs	Brown Trout	17 JUL 2019
BTV_LNF0719_006	Vent	LNF	Livestock Lochs	Brown Trout	17 JUL 2019
BTV_LNF0719_007	Vent	LNF	Livestock Lochs	Brown Trout	17 JUL 2019
BTV_LNF0719_008	Vent	LNF	Livestock Lochs	Brown Trout	17 JUL 2019
BTV_LNF0719_009	Vent	LNF	Livestock Lochs	Brown Trout	17 JUL 2019
BTG_LEV0719_001	Gills	LEU	Livestock Lochs	Brown Trout	11 JUL 2019
BTG_LEV0719_002	Gills	LEU	Livestock Lochs	Brown Trout	11 JUL 2019
BTG_LEV0719_003	Gills	LEU	Livestock Lochs	Brown Trout	11 JUL 2019
BTG_LEV0719_004	Gills	LEV	Livestock Lochs	Brown Trout	19 JUL 2019
BTG_LEV0719_005	Gills	LEV	Livestock Lochs	Brown Trout	19 JUL 2019
BTG_LEV0719_006	Gills	LEV	Livestock Lochs	Brown Trout	19 JUL 2019
BTG_LEV0719_007	Gills	LEV	Livestock Lochs	Brown Trout	19 JUL 2019
BTG_LEV0719_008	Gills	LEV	Livestock Lochs	Brown Trout	19 JUL 2019
BTG_LEV0719_009	Gills	LEV	Livestock Lochs	Brown Trout	19 JUL 2019
BTG_LEV0719_010	Gills	LEV	Livestock Lochs	Brown Trout	19 JUL 2019
BTG_LEV0719_011	Gills	LEV	Livestock Lochs	Brown Trout	19 JUL 2019
BTG_LEV0719_012	Gills	LEV	Livestock Lochs	Brown Trout	19 JUL 2019
BTG_LEV0719_013	Gills	LEV	Livestock Lochs	Brown Trout	19 JUL 2019
BTG_LEV0719_014	Gills	LEV	Livestock Lochs	Brown Trout	19 JUL 2019
BTG_LEV0719_015	Gills	LEV	Livestock Lochs	Brown Trout	19 JUL 2019
BTG_LEV0719_016	Gills	LEV	Livestock Lochs	Brown Trout	19 JUL 2019
BTG_LEV0719_017	Gills	LEV	Livestock Lochs	Brown Trout	19 JUL 2019
BTG_LEV0719_018	Gills	LEV	Livestock Lochs	Brown Trout	19 JUL 2019
BTV_LEV0719_001	Vent	LEU	Livestock Lochs	Brown Trout	11 JUL 2019
BTV_LEV0719_002	Vent	LEU	Livestock Lochs	Brown Trout	11 JUL 2019
BTV_LEV0719_003	Vent	LEU	Livestock Lochs	Brown Trout	11 JUL 2019
BTV_LEV0719_004	Vent	LEV	Livestock Lochs	Brown Trout	19 JUL 2019
BTV_LEV0719_005	Vent	LEV	Livestock Lochs	Brown Trout	19 JUL 2019
BTV_LEV0719_006	Vent	LEV	Livestock Lochs	Brown Trout	19 JUL 2019
BTV_LEV0719_007	Vent	LEV	Livestock Lochs	Brown Trout	19 JUL 2019
BTV_LEV0719_008	Vent	LEV	Livestock Lochs	Brown Trout	19 JUL 2019
BTV_LEV0719_009	Vent	LEV	Livestock Lochs	Brown Trout	19 JUL 2019
BTV_LEV0719_010	Vent	LEV	Livestock Lochs	Brown Trout	19 JUL 2019
BTV_LEV0719_011	Vent	LEV	Livestock Lochs	Brown Trout	19 JUL 2019
BTV_LEV0719_012	Vent	LEV	Livestock Lochs	Brown Trout	19 JUL 2019
BTV_LEV0719_013	Vent	LEV	Livestock Lochs	Brown Trout	19 JUL 2019

BTV_LEV0719_014	Vent	LEV	Livestock Lochs	Brown Trout	19 JUL 2019
BTV_LEV0719_015	Vent	LEV	Livestock Lochs	Brown Trout	19 JUL 2019
BTV_LEV0719_016	Vent	LEV	Livestock Lochs	Brown Trout	19 JUL 2019
BTV_LEV0719_017	Vent	LEV	Livestock Lochs	Brown Trout	19 JUL 2019
BTV_LEV0719_018	Vent	LEV	Livestock Lochs	Brown Trout	19 JUL 2019
BTG_RSA0719_001	Gills	RCA	Sea	Brown Trout	16 JUL 2019
BTG_RSA0719_002	Gills	RCA	Sea	Brown Trout	16 JUL 2019
BTG_SRI0719_001	Gills	SR	Sea	Brown Trout	18 JUL 2019
BTV_RSA0719_001	Vent	RCA	Sea	Brown Trout	16 JUL 2019
BTV_RSA0719_002	Vent	RCA	Sea	Brown Trout	16 JUL 2019
BTV_SRI0719_001	Vent	SR	Sea	Brown Trout	18 JUL 2019
