# Supplementary material for: Absence of Staphylococcus aureus in Wild Populations of Fish Supports a Spillover Hypothesis
Source: Microbiol Spectr. 2023 Jun 21;11(4):e04858-22. doi: 10.1128/spectrum.04858-22 (PMC10434045; doi:10.1128/spectrum.04858-22)
Supplement: Supplemental file 5 — Table S5. Download spectrum.04858-22-s0004.pdf, PDF file, 0.04 MB [file spectrum.04858-22-s0004.pdf]

Table S5: Metadata for all environmental samples collected in Scottish Highlands. The loch code corresponds to names in the Table S3.

Loch code	*Habitat*	*Date of sampling*
LGD	Bird	13_JUL_2019
LCA	Bird	13_JUL_2019
LFE	Bird	13_JUL_2019
LNB	Bird	13_JUL_2019
GRB	Bird	14_JUL_2019
GLO	Isolated	12_JUL_2019
LFO	Isolated	12_JUL_2019
LFI	Isolated	12_JUL_2019
LMG	Isolated	12_JUL_2019
LDD	Isolated	15_JUL_2019
LFM	Isolated	15_JUL_2019
LB	Isolated	15_JUL_2019
LDR	Livestock	11_JUL_2019
LEU	Livestock	11_JUL_2019
LNF	Livestock	17_JUL_2019
LBE	Livestock	19_JUL_2019
LBS	Loch Additional sample	22_JUL_2019
APD	River Additional sample	19_JUL_2019
KED	River Additional sample	22_JUL_2019
SPP	Sea	14_JUL_2019
MUB	Sea	14_JUL_2019
RCA	Sea	16_JUL_2019
SR	Sea	21_JUL_2019
FES	Sea	20_JUL_2019
SBO	Sea	21_JUL_2019
