# Supplementary material for: Absence of Staphylococcus aureus in Wild Populations of Fish Supports a Spillover Hypothesis
Source: Microbiol Spectr. 2023 Jun 21;11(4):e04858-22. doi: 10.1128/spectrum.04858-22 (PMC10434045; doi:10.1128/spectrum.04858-22)
Supplement: Supplemental file 7 — Fig. S1. Download spectrum.04858-22-s0006.pdf, PDF file, 0.03 MB [file spectrum.04858-22-s0006.pdf]

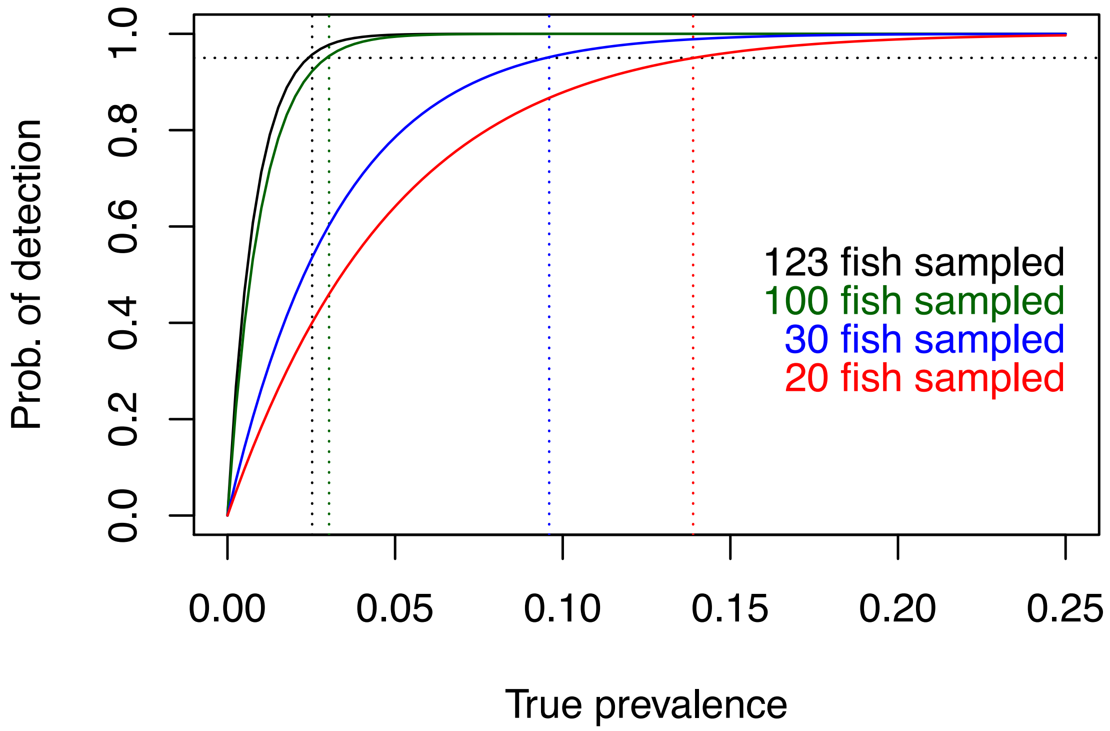

Figure S1: The probability of detecting one or more positive *S. aureus* isolate from fish samples (y axis), given differences in the true prevalence (x axis). The coloured lines correspond to different numbers of fish samples. For example, 20 fish samples gives a 95% chance of detecting one or more positive *S. aureus* isolate if the true prevalence is around 0.14.
